# Correction of the *Caulobacter crescentus* NA1000 Genome Annotation

**DOI:** 10.1371/journal.pone.0091668

**Published:** 2014-03-12

**Authors:** Bert Ely, LaTia Etheredge Scott

**Affiliations:** Department of Biological Sciences, University of South Carolina, Columbia, South Carolina, United States of America; Tel Aviv University, Israel

## Abstract

Bacterial genome annotations are accumulating rapidly in the GenBank database and the use of automated annotation technologies to create these annotations has become the norm. However, these automated methods commonly result in a small, but significant percentage of genome annotation errors. To improve accuracy and reliability, we analyzed the *Caulobacter crescentus* NA1000 genome utilizing computer programs Artemis and MICheck to manually examine the third codon position GC content, alignment to a third codon position GC frame plot peak, and matches in the GenBank database. We identified 11 new genes, modified the start site of 113 genes, and changed the reading frame of 38 genes that had been incorrectly annotated. Furthermore, our manual method of identifying protein-coding genes allowed us to remove 112 non-coding regions that had been designated as coding regions. The improved NA1000 genome annotation resulted in a reduction in the use of rare codons since noncoding regions with atypical codon usage were removed from the annotation and 49 new coding regions were added to the annotation. Thus, a more accurate codon usage table was generated as well. These results demonstrate that a comparison of the location of peaks third codon position GC content to the location of protein coding regions could be used to verify the annotation of any genome that has a GC content that is greater than 60%.

## Introduction

The Sanger sequencing method was developed in 1977 [1], and it remained the primary method of genome sequence analysis for approximately 25 years. The subsequent automation of this method led to many key large-scale accomplishments ranging from the first completed sequence of the 16,569-base pair human mitochondrion in 1981 [2], to first bacterial genome sequence[3], to the completion of the 3 GB human genome [4] which took over a decade to complete. Although Sanger sequencing is considered to be a highly accurate method, it is limited by cost, speed, throughput and scalability. As a result, next-generation technologies emerged that have vastly reduced the time and cost of nucleotide sequencing. Human genomes now can be sequenced in two hours for as little as $1000 in materials [5] and multiple microbial genome sequences can be determined in one day using a single sequencing machine [6], [7]. While the current technologies can generate large amounts of sequence data, it has proven difficult to assemble the sequence data into a finished genome. In November 2013, there were more than 2400 finished and more than 8700 draft bacterial genomes in the IMG database (http://img.jgi.doe.gov/cgi-bin/w/main.cgi). This 3.5-fold difference in draft genomes is likely due to the short reads and lack of paired-end reads for each DNA fragment which are important for orientating and assembling a complete genome [8]. However, even with paired-end reads, it is often difficult to assemble a complete bacterial genome using only short read data [9], [10]. This problem has been solved by the availability of long read data that can be used to accurately place repeated sequences [11] (D. Scott and B. Ely, manuscript in preparation). Thus, the number of finished bacterial genomes is likely to increase dramatically in the near future.

As more genomes are sequenced the need for rapid and inexpensive genome annotation has resulted in a reliance on automated genome analysis and annotation [12]. Automated annotation seeks to identify open reading frames and determine if a given open reading frame codes for a protein based on a particular set of criteria. Once a protein coding region is identified, the amino acid sequence is compared to those in the current database of protein amino acid sequences to determine if it is homologous to proteins of known function. Popular methods for identifying protein coding regions include Glimmer [13], [14], GenemarkHMM [15], [16], and Prodigal [17], and programs which transfer information directly from closely related genomes such as RATT [18] and BG7 [19]. Studies have demonstrated that purely bioinformatics-based pipelines fail to annotate short-length proteins [20], and high G+C content sequences [14], [17], [21]. Other scientists have also found that automated annotation methods lead to the selection of the wrong reading frame, over-annotation of protein coding genes, and incorrect start codon positions, which are all common problems in the microbial genomes deposited in GenBank. For example, E. coli has been found to have ∼500 fewer genes than originally reported [22]. It is estimated that over-annotation is as high as 20% in many genomes [22], [23].

Over-annotation of genomes results from false positive gene detection which means that the genomes contain significant numbers of annotated ORFs that do not code for proteins. These non-coding regions are evident when they overlap a known coding region, but they are more challenging to identify in intergenic regions particularly if they purport to code for small proteins. The annotation of genes coding for large proteins can often be confirmed by matches to homologous genes in other organisms. However, it is much more difficult to identify homologs to small proteins [24], [25] since small regions of amino acid sequence homology occur by chance in unrelated proteins. Another approach used to identify genes that code for proteins is codon usage bias. Codon usage bias is the preferential use of particular codons over others that code for the same amino acid in protein coding regions of DNA. Codon usage bias is greatest in highly expressed genes, whereas genes expressed at very low levels have more uniform levels of codon usage [26], [27]. Therefore it is likely that those ORFs that do not code for proteins have an atypical pattern of codon usage and that this atypical pattern of codon usage could be used to identify inappropriately annotated ORFs. The problem with this approach is that there are genes that are known to code for proteins that also have atypical codon usage patterns. Also, genes that are acquired by horizontal gene transfer from another organism may have patterns of codon usage that are characteristic of that organism but are atypical in their new host genome. Scientists have attempted to overcome these common annotation challenges by using proteomics or RNA sequencing technologies [28]. However, it is not possible to use proteomics to prove that a particular protein is never made. Also, potential coding regions can be transcribed but not translated so the presence of a transcript does not necessarily mean that an open reading frame actually codes for a protein. Therefore, it is important to develop approaches to improve current bacterial genome annotations.

Recently, Yu et al. [29] used a combination of two algorithms to identify 72 and 76 hypothetical genes as non-coding in the genomes of *Pyrococcus horikoshii* and *Caulobacter cresentus* strain CB15, respectively. When we reviewed the results of the *C. crescentus* analysis using a manual inspection of the relevant areas of the genome, we found that we agreed with most of their conclusions. However, we readily identified a number of additional hypothetical genes that probably did not code for proteins. Therefore, although manual re-annotation of microbial genomes is time-consuming, we decided to employ a combination of the computer program MICheck [30] and a manual re-annotation method to improve the accuracy and reliability of the NA1000 genome. We reanalyzed the NA1000 genome because it is the most accurately sequenced and annotated version of the CB15 genome [31], [32], and it is closest to the strain of CB15 that was originally deposited with the ATCC (Melissa Marks, personal communication).

A second problem with current bacterial annotations is that annotation programs often use the first start codon that occurs in an open reading frame. Neilsen et al. [33] reported that up to 60% of annotated prokaryotic genomes contain errors in start/stop codon prediction that can lead to false conclusions about coding sequences and codon usage patterns. To correct this problem, changes in codon usage patterns can help predict the location of the actual start codons in protein coding regions. In organisms with a high genomic GC content such as *C. crescentus*, there is a very high probability that the third codon position will be a G or C, so a third codon position GC content analysis can be used to predict the start of coding regions. Also, comparisons to the amino acid sequence of homologous proteins can be used to predict translation start sites. Therefore, we used a combination of these two approaches to verify the position of the translation start codons in the NA1000 genome.

## Materials and Methods

The annotated genome of *C. crescentus* strain NA1000 (also known as *C. vibiroides* NA1000; Version 23-DEC-2012) was downloaded from GenBank (http://www.ncbi.nlm.nih.gov) and analyzed both with the computer program MICheck (http://www.genoscope.cns.fr/agc/tools/micheck/Form/form.php,) and manually using the computer program Artemis [34]. For the manual annotation, each protein coding sequence (CDS) in the entire genome was examined for third codon position GC content, alignment to a third codon position GC frame plot peak (Fig. 1), and matches in the GenBank database. If a region of the genome included transposase or phage genes and it did not have the host pattern of codon usage, the third codon position peaks of GC content were not observed, and therefore, they could not be used to determine the position of start codons or whether the designated reading frames actually coded for a protein. Therefore, these atypical gene regions were excluded from the analysis. For the remaining genes, if the third codon position GC content was low and lacked a distinct GC peak, and if the deduced amino acid sequence had no significant matches in the Genbank database, the coding sequence was considered to be misannotated and the alternate reading frames were examined to determine if the wrong reading frame had been chosen. An alternate reading frame was considered to be the correct reading frame when the new stop and start codons aligned with the beginning and end of a high third codon position GC peak and when the deduced amino acid sequence of the new peak had significant matches in the Genbank database. If none of the alternate reading frames met these criteria, the gene was considered to be misannotated and was deleted from the annotation. Other coding regions were identified that appeared to be annotated in the correct reading frame, but the annotated reading frame started before the beginning of the high GC peak and the start codons in the matching genes in Genbank were downstream from those used in the current NA1000 annotation. Therefore, the annotation of these genes was modified by the selection of a new start codon that did match the start site of the genes in the database and that also corresponded to the beginning of the GC frame plot peak in the NA1000 annotation.

**Figure 1:**
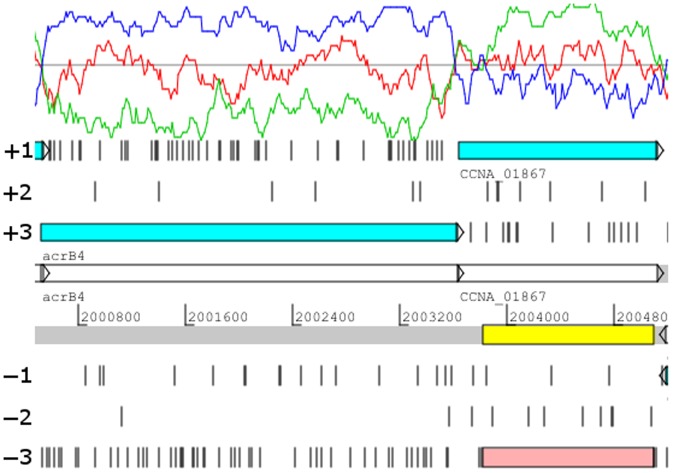
Screen shot of Artemis showing GC Frame Plot and a wrong reading frame. The GC frame plot shows a sliding window of the third codon position GC content for the three forward reading frames. The red line in the GC frame plot corresponds to the +1 reading frame, the green line corresponds to the +2 reading frame and blue line to the +3 reading frame. The three reverse reading frames show the same pattern with the blue, red, green lines corresponding to the −1, −2 and −3 reading frames, respectively. Gene CCNA_1867 (blue bar) is in the wrong reading frame in the 23-DEC-2012 NA1000 annotation. The −3 open reading frame highlighted in pink is the corrected reading frame for gene CCNA_1867.

## Results and Discussion

The annotated genome of *C. crescentus* strain NA1000 (Version 23-DEC-2012) is approximately 4 Mb in size with 3879 genes and a GC content of 67.2%. We analyzed this version of the annotation using MICheck [30] to identify possible instances of misannotation. In addition, since NA1000 has a high GC content, we were able to assess the quality of the 23-DEC-2012 version of the annotation by using the GC frame plot feature in Artemis [34]. Most NA1000 protein coding regions have high third codon position GC content, and the graphical output of the GC frame plot makes it easy to distinguish a protein-coding reading frame from a non-coding reading frame. For example, the *acrB4* gene is correctly annotated in the +3 reading frame that starts and ends at the boundaries of the third codon position high GC peak (Fig.1). In contrast, the alternative minus-2 open reading frame starts at the beginning of the high third position GC peak but terminates well before the end of the high GC peak so it is not likely to code for a protein. With the aid of GC frame plot, each CDS in the 23-DEC-2012 version of the annotation was manually evaluated and considered to be a true protein-coding gene if the start and stop codons corresponded to the beginning and end of a GC frame plot peak, respectively, and if the reading frame did not overlap an adjacent CDS more than a few bases. More than 90% of CDS examined met these criteria and were considered to be accurately identified protein coding genes using both MICheck and the manual method. In addition, both methods identified large numbers of misannotated genes. The two lists of misannotated genes had considerable overlap but each method was able to identify significant numbers of misannotated genes that had been overlooked by the other method.

In contrast to the *acrB4* gene, the positions of 38 genes did not align with a third codon position GC peak and had no significant database matches. However, in each case there was an alternative reading frame that did have a high third codon position GC content that was aligned with a set of start and stop codons (Table 1). For example CCNA_01867 was originally thought to code for a protein in the +1 reading frame with no match in the GenBank database (Fig. 1). However, the highest third codon position GC peak corresponds to the -3 reading frame, not the +1 reading frame. This inconsistency led us to identify the -3 open reading frame (Fig. 1 pink bar) as an alternative reading frame. When the amino acid sequence of this alternative reading frame was compared to those in the GenBank database, more than 50 significant matches to a highly conserved sugar transport protein gene were obtained. Therefore, we concluded that the -3 reading frame was the correct reading frame. Similar results were obtained for the other 37 genes listed in Table 1. Of the 38 newly identified reading frames, 26 had strong matches to previously annotated genes in other species of *Caulobacter* and five of these 26 matches were to genes coding for proteins with known functions. Most of the remaining newly identified reading frames coded for proteins that matched proteins from other species of bacteria that are closely related to *Caulobacter*. Thus there is strong evidence in each of these 38 cases that the correct reading frame had not been identified in the original NA1000 annotation.

**Table 1:** *C. crescentus* NA1000 genes with a changed reading frame.

Gene	Genome Coordinates*	Gene product	Source of matching genes
CCNA_00513	527386..527931	Conserved hypothetical protein	non caulobacter
CCNA_00581	609290…611077c	Conserved hypothetical protein	non caulobacter
CCNA_00599	635020…635670	Conserved hypothetical protein	Caulobacter
CCNA_00702	761965…762321	Conserved hypothetical protein	Caulobacter
CCNA_00713	770295…770444	Hypothetical protein	Caulobacter
CCNA_00786	850119…850442c	Hypothetical protein	Caulobacter
CCNA_00868	946717…947265	Conserved hypothetical protein	Caulobacter
CCNA_01127	1231609…1232985	Conserved hypothetical protein	Caulobacter
CCNA_01150	1254292…1254639c	Conserved hypothetical protein	Caulobacter
CCNA_01265	1395129..1395539c	Transposase	non caulobacter
CCNA_01293	1418452..1418862	Transposase	non caulobacter
CCNA_01411	1529256…1529711	Conserved hypothetical protein	Caulobacter
CCNA_01435	1549757…1550008c	Transglycosylase associated protein	Caulobacter
CCNA_01518	1627542…1628183c	Metal dependent phosphohydrolase	Caulobacter
CCNA_01720	1848134…1848523	Conserved hypothetical protein	Caulobacter
CCNA_01867	2003818…2005050	Conserved hypothetical protein	Caulobacter
CCNA_01871	2009957…2010232c	Hypothetical protein	non significant
CCNA_02079	2230242…2230706	Conserved hypothetical protein	Caulobacter
CCNA_02114	2263326..2263493c	Hypothetical protein	non significant
CCNA_02168	2321658…2321954c	Conserved hypothetical protein	Caulobacter
CCNA_02323	2465238..2465669	No database match	non significant
CCNA_02393	2536532…2536966	Limonene-1,2-epoxide hydrolase	Caulobacter
CCNA_02524	267256…2673444c	Conserved hypothetical protein	non caulobacter
CCNA_02536	2684042…2684413	Conserved hypothetical protein	Caulobacter
CCNA_02585	2733698…2734024	Conserved hypothetical protein	non caulobacter
CCNA_02871	3021200..3021598c	Gene transfer agent (GTA)-like protein	Caulobacter
CCNA_02880	3027016..3028377c	Phage DNA packaging protein	Caulobacter
CCNA_02968	3125241…3125573c	Conserved hypothetical protein	non caulobacter
CCNA_02990	3144126…3144536	Transposase	non caulobacter
CCNA_02998	3153124…3153330	Conserved hypothetical protein	Caulobacter
CCNA-03251	3422211..3423167c	MarR family transcriptional regulator	Caulobacter
CCNA_03361	3539803…3540480c	Conserved hypothetical protein	non caulobacter
CCNA_03411	3578916…3579266	Conserved hypothetical protein	Caulobacter
CCNA_03427	3592615…3592957c	Conserved hypothetical protein	Caulobacter
CCNA_03470	3636805…3637563c	Conserved hypothetical protein	Caulobacter
CCNA_03566	3720403..3720825c	Conserved hypothetical protein	Caulobacter
CCNA_03654	3815982…3816086	Conserved hypothetical protein	Caulobacter
CCNA_03785	3952640..3953299	Conserved hypothetical protein	Caulobacter

*A lower case “c” indicates that the coding sequence is on the complementary strand of the DNA.

In three of the 38 cases described above, CCNA_02393, CCNA_02871, and CCNA_02968, we also identified a second open reading frame where there was a clear high third codon position GC peak that did not correspond to a previously annotated gene or overlap with the adjacent genes. When the predicted amino acid sequences of these open reading frames were compared to those in the GenBank database, there were significant matches to a metallo-bactalactamase, a phage protein, and a conserved hypothetical protein (Table 2). In addition, we identified eight other previously overlooked open reading frames in other parts of the NA1000 genome that had significant database matches (Table 2). Therefore, we concluded that each of these eleven regions coded for a protein, and we added them to the NA1000 annotation.

**Table 2:** New predicted genes.

Temporary Gene ID	Gene Position*	Predicted Gene Function
CCNA_01158B	1264353..1264793	Sugar Translocase
CCNA_01340B	1452580..1453071	Activator of Hsp90 ATPase 1-like protein
CCNA_01547B	1658853..1659293c	Conserved hypothetical
CCNA_02123B	2274650..2275060	Transposase
CCNA_02393B	2537009..2537347	Metallo-bactalactamase
CCNA_02648B	2801810..2802232	Hypothetical protein
CCNA_02871B	3021598..3021903	Phage packaging-like protein
CCNA_02880B	3028202..3028606c	Conserved hypothetical
CCNA_02968B	3125573..3125986c	Conserved hypothetical
CCNA_03112B	3263371..3263808	Conserved hypothetical
CCNA_03080B	3228849..3229025c	Oligosaccharyl transferase subunit (alpha)

*A lower case “c” indicates that the coding sequence is on the complementary strand of the DNA.

There were numerous additional genes that did not align with a third codon position GC peak and had no significant matches to any genes in the GenBank database. Many of these genes were in regions that included mobile elements, phages, or other insertions and had low third codon position GC content. Since most of these regions had a reduced overall GC content, the protein-coding genes in these regions would not be expected to align with a high third codon position GC peak, and therefore no changes to the annotation of the genes in these regions were proposed. However, we did identify 112 genes in the current annotation that were not associated with mobile elements or other low GC regions of the genome and also did not align with a third codon position GC peak (Table 3 and Table S1). These genes also did not have significant matches to any genes in the GenBank database. Therefore, we propose that these 112 genes do not code for a protein and should be removed from the 23-DEC-2012 annotation. This conclusion is consistent with the data of Christen et al. [32] and Fang et al. [35], which showed that none of the 112 genes coded for essential proteins.

**Table 3:** Deletion of previously annotated genes.

Gene	Gene Position*	Number of deleted codons
CCNA_00242	256209..256364c	51
CCNA_00258	271669..271875	68
CCNA_00289	300549..300845c	98
CCNA_00325	338371..338499	42
CCNA_00347	361909..362088	59
CCNA_00409	424307..424450	47
CCNA_00418	430572..430736	54
CCNA_00577	601417..601599c	60
CCNA_00584	612811..613236	141
CCNA_00606	642000..642233	77
CCNA_00739	79853..798380c	43
CCNA_00771	826360..826482	40
CCNA_00797	861100..861297c	65
CCNA_00816	879721..879912c	63
CCNA_00819	882074..882181c	35
CCNA_00829	892673..892915c	80
CCNA_00848	921330..921674c	114
CCNA_00877	954339..954947c	202
CCNA_00896	975549..975920c	123
CCNA_00949	1026741..1026965	74
CCNA_00955	1032152..1032286c	44
CCNA_00960	1038046..1038144	32

*A lower case “c” indicates that the coding sequence is on the complementary strand of the DNA.

After two of the 112 genes, CCNA_00584 and CCNA_02119, were deleted, we realized that in both cases the location of the third position GC peak of the adjacent gene suggested that it probably used an upstream start codon. This hypothesis was confirmed by a BLAST analysis that showed a homologous region upstream of the previously annotated start codon and a new start codon was chosen that better fit the GC peak and was consistent with that of the database matches. Thus we added 573 nucleotides to CCNA_00583 and 639 nucleotides to CCNA_02118, adding 191 amino acids to the hypothetical protein coded by CCNA_00583 and 213 amino acids to the ATP-dependent helicase protein coded by CCNA_02118.

Although in the two cases described above the annotated genes were shorter than the actual genes, we found more than 100 instances where the annotated gene was too long, and that beginning the gene with a downstream start codon was more appropriate. When the alignment of the annotated genes was compared with the position of the high GC third codon regions, we found 111 genes where the currently defined coding region was in the correct reading frame, but the reading frame started before the beginning of the high GC third codon position peak (Table 4 and Table S2). When the predicted amino acid sequence of these 111 genes was compared to those of the homologous genes in the GenBank database, the start codons in the homologous genes were downstream from those used in the NA1000 current annotation. For example, when CCNA_00338 was compared to the GenBank database, the amino acid sequences of the matching proteins started approximately 78 amino acids downstream from the first amino acid in the original CCNA_00338 annotation. Therefore, a new start codon 234 bases downstream of the original start codon was chosen to match the start site of the homologues in the database. Significantly, the new start codon corresponded to the beginning of the high GC frame plot peak in the NA1000 annotation whereas the original annotation of the coding region started prior to the high third position GC peak and overlapped the coding region of CCNA_0037 (white box in Fig. 2). Thus, the comparison of the coding regions to the corresponding high GC peak allowed us to confirm and correct the start codon of the CCNA_00338. A similar approach was utilized for the remaining 110 genes that were shortened (Table 4 and Table S2).

**Figure 2:**
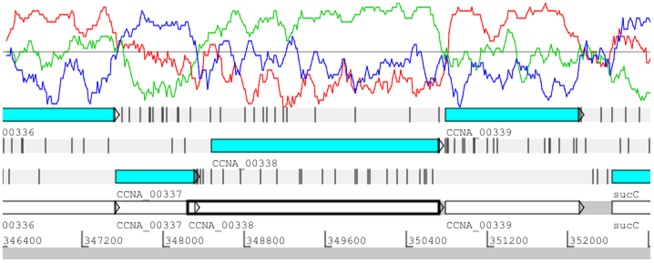
Screen shot of Artemis showing the correction of two overlapping gene annotations. The CCNA_00338 gene in the +2 reading frame has been shortened relative to the open reading frame white box that corresponds to the original CCNA_00338 reading frame. Note that the original start site was upstream of the CCNA_00337 stop codon.

**Table 4:** Genes with modified start sites.

Gene	Gene Position*	Modified Gene Position	Gene Function
CCNA_00156	164586..164951	164685..164951	ArsR family transcriptional regulator
CCNA_00176	191399.191956	191468..191956	Type II secretion pathway protein H
CCNA_00177	191925..192308	191937..192308	General secretion pathway protein I
CCNA_00230	245765..247030c	245765..247003c	Ribosomal large subunit pseudouridine synthase B
CCNA_00304	318031..319308c	318031..319263c	3-deoxy-D-manno-octulosonic-acid transferase
CCNA_00318	333101..334138	333179..334138	Hypothetical protein
CCNA_00338	348245..350719	348479..350719	TonB-dependent receptor
CCNA_00438	444889..445263c	444889..445200c	Hypothetical protein
CCNA_00465	477921..479033	477936..479033	UDP-galactopyranose mutase
CCNA_00481	497307..497597	497313..497597	HipB transcriptional regulator
CCNA_00582	611119..611757	611257..611757	Hypothetical protein
CCNA_00613	654176..655519c	654176..655399c	Cyanophycinase
CCNA_00641	692376..692672c	692376..692645c	Hypothetical protein
CCNA_00656	710639..712531	710696..712531	Type I restriction-modification system, M subunit
CCNA_00661	718799..719233c	718799..719176c	Transposase
CCNA_00690	747704..748261c	747704..748207c	CarD-like transcriptional regulator
CCNA_00756	813842..814120c	813842..814018c	Hypothetical protein
CCNA_00772	827021..827428c	827021..827239c	Hypothetical protein
CCNA_00860	938619..938855c	938622..938825c	Hypothetical protein
CCNA_00884	963806..964192	963806..964180c	Hypothetical protein

*A lower case “c” indicates that the coding sequence is on the complementary strand of the DNA.

Once the review of the current annotation was completed, we realized that the changes in the annotated reading frames that resulted in the removal of regions that did not actually code for proteins might remove a significant fraction of the genes containing atypical codon usage patterns. As a result, we predicted that there would be a reduction in the frequency of rarely used codons in a revised codon usage table that was based on the improved annotation. Therefore, a new codon usage table from the updated annotation of NA1000 was derived using the Artemis program. For this comparison, codons were considered to be rare codons if the relative occurrence of the codon was less than 10 per thousand in the codon usage table derived from the 23-DEC-2012 version of the annotation. Using this criterion, we identified 33 rarely used codons and found that the relative occurrence decreased for 26 out of these 33 rare codons when the old and new codon usage tables were compared (Fig. 3). Six of the other seven rare codons were used at the same frequency and one, UAU, was used slightly more frequently. Conversely, the relative occurrence of 16 out of the 28 more commonly used codons increased and seven others stayed the same (Fig. 3). In the remaining five commonly used codons, the frequency went down but the frequency of the most common codon of that codon family increased in each case. This reduction in the use of common, but second choice codons would be consistent with expected changes resulting from the removal of noncoding regions from the annotation. In the remaining case, the frequency of both glutamate codons decreased indicating that glutamate codons were over-represented in the regions that are no longer considered coding regions. Thus these results are consistent with the idea that the two methods of identifying protein-coding genes allowed us to remove non-coding regions that had atypical codon usage patterns.

**Figure 3:**
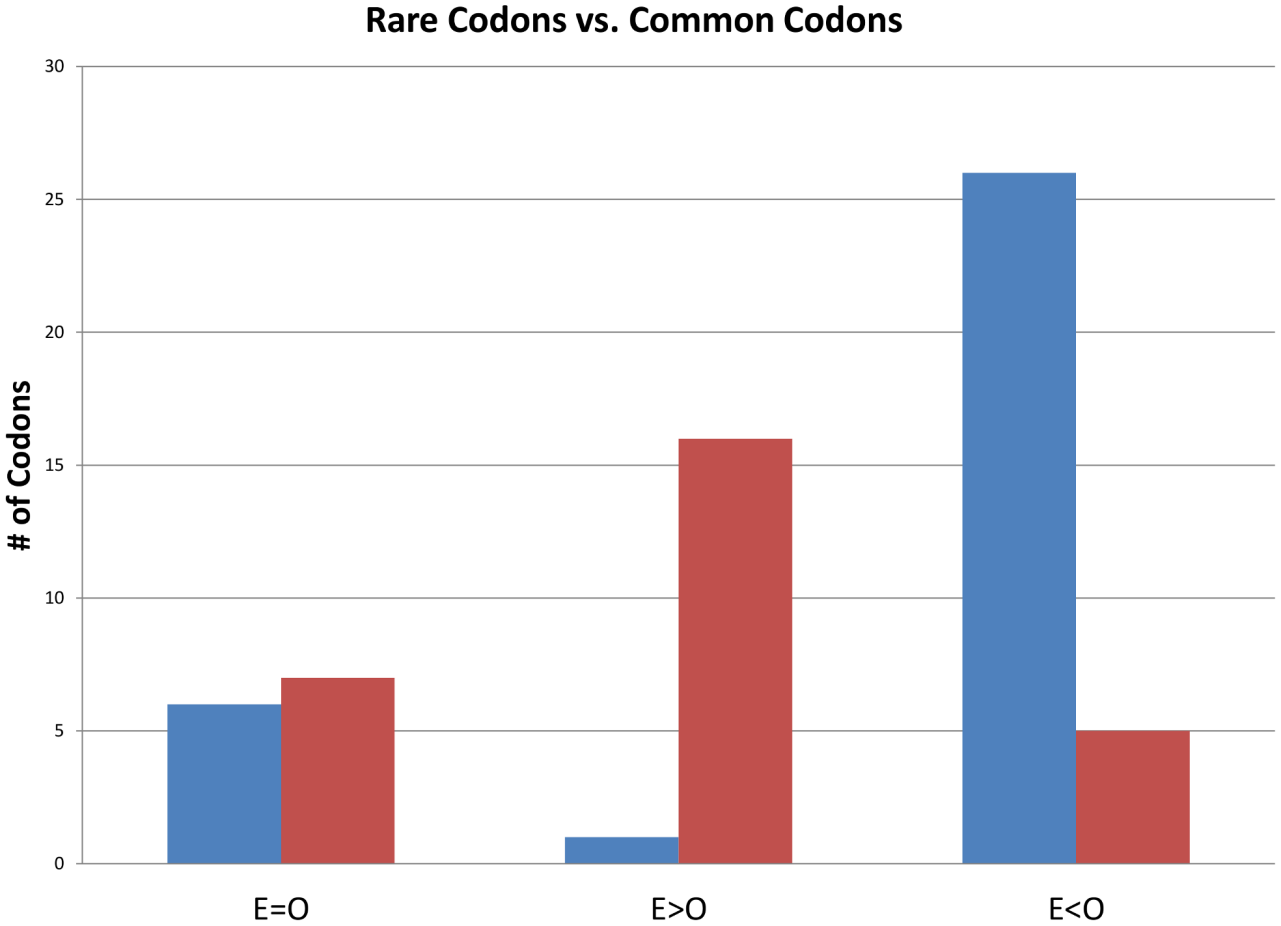
Comparison of the frequency of rare codons and common codons in the original (O) and edited (E) NA1000 genome annotations. The blue bar represents the number of rare codons, and the red bar represents the number of common codons that have a codon usage frequency in the edited genome annotation (E) that is equal to, greater than, or less than the frequency in the original genome (O) annotation. Nonsense codons were excluded from this analysis.

In this study, we demonstrated that a combination of a manual inspection with an automated evaluation of the *C. crescentus* genome annotation using MICheck resulted in the identification of more than 200 errors in the existing annotation. Each evaluation method found annotation errors that were not identified by the other method. Therefore, it appears that our manual approach checks for patterns based on third position GC content that are not assessed by MICheck. However, MICheck was able to identify annotation errors that our manual approach should have detected but they escaped the attention of our human analysis. This problem with the manual analysis could be corrected by automating our manual pattern recognition approach. The program would first calculate the third position GC content for each of the six possible reading frames excluding regions with low overall GC content, and then, compare the positions of the regions of high third position GC content to the positions of the annotated coding regions and generate a file of regions where a one to one correspondence was absent. If no annotated coding regions were detected opposite a high third position GC peak, the open reading frames (ORFs) in the region would be examined for an appropriate match. If a matching ORF was identified, the corresponding amino acid sequence would be compared to the NCBI database using BLAST, and the presence of significant matches in the database would verify that the ORF coded for a protein. Similarly if no high third position GC peak was present for a particular annotated coding region and the flanking genes did have high third position GC peaks, the corresponding amino acid sequence would be compared to the NCBI database using BLAST, and the absence of significant matches in the database would suggest that the ORF was unlikely to code for a protein. In cases where the high third position GC peak was downstream from the beginning of the annotated coding region, the corresponding amino acid sequence also would be compared to the NCBI database using BLAST, and the positions of the first amino acids of the database matches would be compared to that of the annotated gene. If the annotated gene contained a second start codon that corresponded to the one used in the database matches, the annotation would be changed to use the alternate start codon. This type of automated analysis of the location of the high third position GC peaks relative to the designated protein coding regions would save a considerable amount of time and eliminate the human errors that arise from a manual analysis.

In summary, we used an analysis of third codon position GC content to improve the accuracy of the *C. crescentus* NA1000 genome annotation. We identified 11 new genes, modified the start site of 113 genes, changed the reading frame of 38 misidentified genes, and removed 112 non-coding regions that had been designated as coding regions. We have observed that high third position GC peaks are present in genomes with an overall GC content of 60%. Therefore, an analysis of the location of the high third position GC peaks with respect to the position of protein coding open reading frames could be used to verify the genome annotation for any species with a genomic GC content that is greater than 60%.

## Supporting Information

Table S1 **Genes deleted from the** ***C. crescentus*** **NA1000; Version 23-DEC-2012 annotation.**
(DOCX)

Table S2 **Genes that were shortened from the** ***C. crescentus*** **NA1000; Version 23-DEC-2012 annotation.**
(DOCX)
