# Supplementary material for: Correction of the Caulobacter crescentus NA1000 Genome Annotation
Source: PLoS One. 2014 Mar 12;9(3):e91668. doi: 10.1371/journal.pone.0091668 (PMC3951458; doi:10.1371/journal.pone.0091668)
Supplement: Table S1 — Genes deleted from the C. crescentus NA1000; Version 23-DEC-2012 annotation. (DOCX) [file pone.0091668.s001.docx]

Table S1. Genes deleted from the *C. crescentus* NA1000; Version 23-DEC-2012 annotation.

CCNA_00242

CCNA_00258

CCNA_00289

CCNA_00325

CCNA_00347

CCNA_00409

CCNA_00418

CCNA_00577

CCNA_00584

CCNA_00606

CCNA_00739

CCNA_00771

CCNA_00797

CCNA_00816

CCNA_00819

CCNA_00829

CCNA_00848

CCNA_00877

CCNA_00896

CCNA_00949

CCNA_00955

CCNA_00960

CCNA_00964

CCNA_00975

CCNA_01043

CCNA_01126

CCNA_01146

CCNA_01160

CCNA_01304

CCNA_01331

CCNA_01370

CCNA_01377

CCNA_01401

CCNA_01402

CCNA_01428

CCNA_01436

CCNA_01454

CCNA_01455

CCNA_01456

CCNA_01482

CCNA_01540

CCNA_01544

CCNA_01561

CCNA_01565

CCNA_01631

CCNA_01661

CCNA_01662

CCNA_01784

CCNA_01796

CCNA_01798

CCNA_01838

CCNA_01839

CCNA_01861

CCNA_01862

CCNA_01872

CCNA_01887

CCNA_01910

CCNA_02004

CCNA_02009

CCNA_02038

CCNA_02107

CCNA_02114

CCNA_02119

CCNA_02147

CCNA_02148

CCNA_02162

CCNA_02248

CCNA_02276

CCNA_02286

CCNA_02527

CCNA_02532

CCNA_02533

CCNA_02551

CCNA_02582

CCNA_02707

CCNA_02796

CCNA_02799

CCNA_02857

CCNA_02890

CCNA_02911

CCNA_02926

CCNA_02928

CCNA_02932

CCNA_02952

CCNA_02958

CCNA_03014

CCNA_03015

CCNA_03034

CCNA_03109

CCNA_03145

CCNA_03150

CCNA_03220

CCNA_03237

CCNA_03264

CCNA_03274

CCNA_03275

CCNA_03286

CCNA_03307

CCNA_03329

CCNA_03472

CCNA_03525

CCNA_03526

CCNA_03542

CCNA_03550

CCNA_03551

CCNA_03576

CCNA_03606

CCNA_03608

CCNA_03638

CCNA_03662

CCNA_03720

CCNA_03829
