# Supplementary material for: Correction of the Caulobacter crescentus NA1000 Genome Annotation
Source: PLoS One. 2014 Mar 12;9(3):e91668. doi: 10.1371/journal.pone.0091668 (PMC3951458; doi:10.1371/journal.pone.0091668)
Supplement: Table S2 — Genes that were shortened from the C. crescentus NA1000; Version 23-DEC-2012 annotation. (DOCX) [file pone.0091668.s002.docx]

Table S2. Genes that were shortened from the *C. crescentus* NA1000; Version 23-DEC-2012 annotation.

| 1. CCNA_00156 2. CCNA_00176 3. CCNA_00177 4. CCNA_00230 5. CCNA_00304 6. CCNA_00318 7. CCNA_00338 8. CCNA_00438 9. CCNA_00465 10. CCNA_00481 11. CCNA_00582 12. CCNA_00613 13. CCNA_00641 14. CCNA_00656 15. CCNA_00661 16. CCNA_00690 17. CCNA_00756 18. CCNA_00772 19. CCNA_00860 20. CCNA_00884 21. CCNA_00927 22. CCNA_01014 23. CCNA_01015 24. CCNA_01092 25. CCNA_01116 26. CCNA_01154 27. CCNA_01158 28. CCNA_01211 29. CCNA_01218 30. CCNA_01231 31. CCNA_01237 32. CCNA_01250 33. CCNA_01247 34. CCNA_01283 35. CCNA_01284 36. CCNA_01340 37. CCNA_01347 38. CCNA_01348 39. CCNA_01374 40. CCNA_01423 41. CCNA_01452 42. CCNA_01722 43. CCNA_01760 44. CCNA_01779 45. CCNA_01886 46. CCNA_01993 47. CCNA_03501 48. CCNA_03514 49. CCNA_03563 50. CCNA_03612 51. CCNA_03627 52. CCNA_03643 53. CCNA_03705 54. CCNA_03710 55. CCNA_03716 56. CCNA_03728 57. CCNA_03743 58. CCNA_03752 59. CCNA_03754 60. CCNA_03799 61. CCNA_03804 62. CCNA_03829 63. CCNA_03833 64. CCNA_03864 65. CCNA_03872 | 164685..164951  191468..191956  191937..192308  245765..247003c*  318031..319263c  333179..334138  348479..350719  444889..445200c  477936..479033  497313..497597  611257..611757  654176..655399c  692376..692645c  710696..712531  718799..719176c  747704..748207c  813842..814018c  827021..827239c  938622..938825c  963806..964180c  1003804..1004187c  1094791..1095267c  1095316..1096998  1198379..1198669c  1220535..1222292  1257902..1258591  1264793..1266709  1337787..1338569c  1344656..1345570  1358768..1359754  1363332..1363481c  1377436..1378422  1374356..1375792c  1409671..1410771  1410768..1411604  1453071..1454321  1460382..1461083  1461555..1462034  1488315..1490177  1539478..1540509  1565904..1566356  1849126..1849506  1887107..1889506c  1906532..1906795c  2025426..2028197c  2139697..2140893c  3658588..3659460c  3671916..3672518c  3718691..3719245c  3767028..3767276c  3782612..3783742  3802816..3803235c  3873755..3874225  3876441..3876905c  3881363..3881965  3896761..3897033c  3912262..3913677c  3921707..3922582  3923690..3924376c  3962492..3963331c  3969955..3970242c  3994198..3994335c  3997994..3998809c  4025504..4026016  4034962..4036302c |  | 1. CCNA_02016 2. CCNA_02019 3. CCNA_02163 4. CCNA_02183 5. CCNA_02219 6. CCNA_02256 7. CCNA_02258 8. CCNA_02297 9. CCNA_02311 10. CCNA_02316 11. CCNA_02319 12. CCNA_02331 13. CCNA_02434 14. CCNA_02473 15. CCNA_02598 16. CCNA_02695 17. CCNA_02713 18. CCNA_02744 19. CCNA_02746 20. CCNA_02784 21. CCNA_02808 22. CCNA_02863 23. CCNA_02871 24. CCNA_02880 25. CCNA_02979 26. CCNA_02891 27. CCNA_03021 28. CCNA_03022 29. CCNA_03079 30. CCNA_03080 31. CCNA_03081 32. CCNA_03102 33. CCNA_03119 34. CCNA_03162 35. CCNA_03169 36. CCNA_03175 37. CCNA_03259 38. CCNA_03266 39. CCNA_03270 40. CCNA_03298 41. CCNA_03314 42. CCNA_03321 43. CCNA_03333 44. CCNA_03397 45. CCNA_03435 46. CCNA_03457 | 2162766..2164250c  2166636..2167253c  2317527..2318078  2334262..2335152  2365481..2366239  2407281..2408717c  2409438..2410118  2444280..2444810c  2457093..2457431  2460427..2460870c  2461698..2462000c  2473027..2473827  2575252..2575692c  2616200..2619331  2744972..2745238c  2849457..2849675  2871850..2873037  2906466..2907182c  2908313..2909275  2942275..2943096  2963521..2963979  3016985..3017470c  3021200..3021598c  3027016..3028236c  3134421..3135581c  3038365..3039954  3175645..3175830c  3175827..3176915c  3228294..3228587c  3228577..3228828c  3229064..3229510c  3252367..3253821c  3267484..3267882c  3317963..3319363c  3327940..3328524  3334806..3335162c  3429300..3429599c  3436670..3437032c  3440693..3441151  3469206..3472199c  3488838..3489665  3502114..3502482c  3512269..3514200c  3568610..3568840c  3598673..3600010  3620488..3621168c |
| --- | --- | --- | --- | --- |

*A lower case “c” indicates that the coding sequence is on the complementary strand of the DNA.
